# Retinal nerve fiber layer changes in migraine: A protocol for systematic review and meta-analysis

**DOI:** 10.1097/MD.0000000000021680

**Published:** 2020-08-14

**Authors:** XiaoGuang Lin, ZhongQuan Yi, XueLing Zhang, QinQin Liu, RuYuan Cai, ChaoChun Chen, HongJie Zhang, PanWen Zhao, PingLei Pan

**Affiliations:** a Department of Neurology, Affiliated Suqian Hospital of Xuzhou Medical University, Suqian; b Department of Central Laboratory; c Department of Ophthalmology; d Department of Neurology, Affiliated Yancheng Hospital, School of Medicine, Southeast University, Yancheng, PR China.

**Keywords:** migraine, retinal nerve fiber layer, optical coherence tomography, migraine without aura, migraine with aura

## Abstract

**Background:**

Migraine is a common neurological disease, which seriously affects the quality of life and daily activities of patients. Although migraine is a transient phenomenon of cerebral vasoconstriction, it is well documented that recurrent attacks of migraine may lead to abnormalities in retinal structure. Optical coherence tomography (OCT) is a sensitive method to detect subtle damage in retinal nerve fiber layer (RNFL). There have been many studies investigating the difference in RNFL thickness with optical coherence tomography (OCT) between migraine patients and healthy controls. However, the results were not consistent. Our purpose is to perform a meta-analysis to investigate RNFL alterations in migraine.

**Methods:**

We will search PubMed, Embase, Web of science for studies assessing the differences in RNFL measured by OCT between patients with migraine and healthy controls. Case-control studies published in English will be included. Two reviewers will independently screen eligible articles, extract data, and assess quality. This meta-analysis will synthesize selected research data and compare the difference in RNFL thickness between patients with migraine and healthy controls. We will use Stata 15 in this meta-analysis. I^2^ statistics will be used to assess heterogeneity. If I^2^ ≤ 50%, the data are synthesized will use a fixed effect model. Otherwise, a random effect model will be performed. Publication bias will be determined by the Egger test. The methodological quality of all included studies will be evaluated by the Newcastle-Ottawa Scale (NOS). We will perform subgroup analysis, sensitivity analysis, and meta-regression analysis to test the robustness of the results.

**Results:**

We will obtain quantitative results regarding the difference in RNFL thickness between migraine patients and healthy controls. The results will be published in a peer-reviewed journal.

**Conclusions:**

The results of this study provide a high-quality synthesis of existing evidence and provide a basis for assessing the effect of migraine on the thickness of RNFL.

**Registration number:**

INPLASY 202060033

## 1 Introduction

According to World Health Organization data, migraine has become the third most common disease in the 21st century.^[1]^ Meanwhile, migraine is one of the top 10 causes of disability in the world.^[2,3]^ Migraine has generally increased in incidence worldwide in recent years, especially in developing countries, possibly with adverse lifestyle changes brought about by rapid urbanization in these regions.^[4–6]^ Migraine is characterized by moderate to severe headache with fatigue, depression, hyperactivity, nausea, sensitivity to light or sound and other neurological symptoms.^[7,8]^ The international classification of headaches divides migraine into 2 main types: migraine without aura and migraine with aura.^[9]^ There is no consensus on the pathogenesis of migraine in the medical community, but it is generally accepted that migraine is caused by the combined involvement of nerves and blood vessels.^[10–12]^ Cortical spreading depression (CSD) has been known to play an important role in the pathogenesis of migraine, which can activate and sensitize the trigeminal vascular system (TGVS), then triggers migraine-associated neurological and vascular responses, and finally induces pain.^[11,13]^ Although migraine is a transient phenomenon of cerebral vasoconstriction, the chronicity of migraine may lead to retinal structural abnormalities.^[14,15]^ There is evidence that ganglion cell death in migraine patients may be secondary to alterations in the microcirculation of the optic nerve head or even in the quality of retinal perfusion.^[16,17]^ Optical coherence tomography (OCT) is a rapid, reproducible, and economical imaging technique for high-resolution quantitative assessment of the retina and retinal nerve fiber layer (RNFL).^[18–20]^ Similar to intravascular ultrasound, OCT uses near-infrared light to generate cross-sectional vascular images and it has been shown to be reliable and reproducible. Since the official commercialization of OCT technology in 2002, an alarming number of literatures have used OCT technology to study optic neuropathy. OCT have been developed to help diagnose neuro-ophthalmic diseases and detect disease development.^[21,22]^ OCT technology continues to evolve, and spectral-domain OCT (SD-OCT) has replaced time-domain OCT (TD-OCT) as the first choice for ophthalmic OCT instruments.^[23]^ In contrast to TD-OCT, SD-OCT uses ultra-fast frequency scanning light source, with faster scanning speed, higher sensitivity, and superior high resolution, especially for more accurate segmentation of the retinal layer.^[24–26]^

In recent years, many studies have evaluated the changes of RNFL thickness in patients with migraine compared with healthy subjects. Most studies reported a decrease of RNFL thickness in migraines,^[27,28]^ while only a few articles yielded an increase of RNFL thickness.^[29,30]^ A previous meta-analysis evaluated the relationship between migraine and OCT-measured RNFL thickness, but we also found some issues worthy of further exploration.^[31]^ First, the number of included studies was 6 and the latest one included in their meta-analysis was published in 2014. Since then, more eligible studies regarding this topic have been published. Second, the influence of OCT instruments on RNFL thickness measurements was not analyzed. As mentioned earlier, SD-OCT is able to segment the retinal layer more accurately than TD-OCT. RNFL measurement data are affected by the OCT instrument.^[32]^ Third, due to the small number of studies, regression analysis was not performed to investigate the potential effects of age, gender, disease duration, attack frequency, pain intensity, and intraocular pressure on RNFL. Therefore, we will perform an update meta-analysis, and add new subgroup analyses and regression analyses to re-evaluate the relationship between RNFL thickness and migraine.

## 2 Methods

The protocol of this study was registered on the International Platform of Registered Systematic Review and Meta-Analysis Protocols (INPLASY) and the registration number is INPLASY202060033 (URL = https://inplasy.com/inplasy-2020-6-0033/). The preferred reporting items for systematic reviews and meta-analysis protocols (PRISMA-P) statement was the guideline during the design of this study.^[33]^

### 2.1 Eligibility criteria for study selection

#### 2.1.1 Types of studies

We will only select case-control studies using OCT to measure the RNFL thickness in migraine and healthy controls. The language included in the literature is limited to English. Animal studies, abstracts, letters, reviews and case-studies will be excluded.

#### 2.1.2 Types of participants

We will include articles on patients older than 18 years with normal visual fields who have been diagnosed with migraine according to the International Headache Disease Classification. Patients will be excluded if they meet the following exclusion criteria:

(1) incorporate any form of glaucoma, optic nerve disease, or intraocular surgical intervention;
(2) diabetes with evidence of retinopathy such as hemorrhage, hard and/or soft exudate, macular edema;
(3) neurological disorders that may affect RNFL thickness;
(4) children (under 18 years of age).

#### 2.1.3 Types of interventions

We will include all studies that use optical coherence tomography (OCT) to evaluate the effects of migraine on the thickness of the mean and segmental RNFL. The control group will use healthy people who do not suffer from migraine.

#### 2.1.4 Types of outcomes

The main outcome of this review is the difference in mean RNFL thickness and segmental RNFL thickness between migraine patients and health controls.

### 2.2 Search method and strategy

We will search the electronic databases PubMed, EMBASE and Web of Science. The primary search strategies are: (“optical coherence tomography” OR “retina nerve fiber layer” OR “RNFL”) AND “migraine”. The date of the last search is set at 18 March, 2020. Additionally, the reference lists of relevant reviews and the articles selected for inclusion will be manually searched.

### 2.3 Data collection and analysis

#### 2.3.1 Study selection

**Figure 1:**
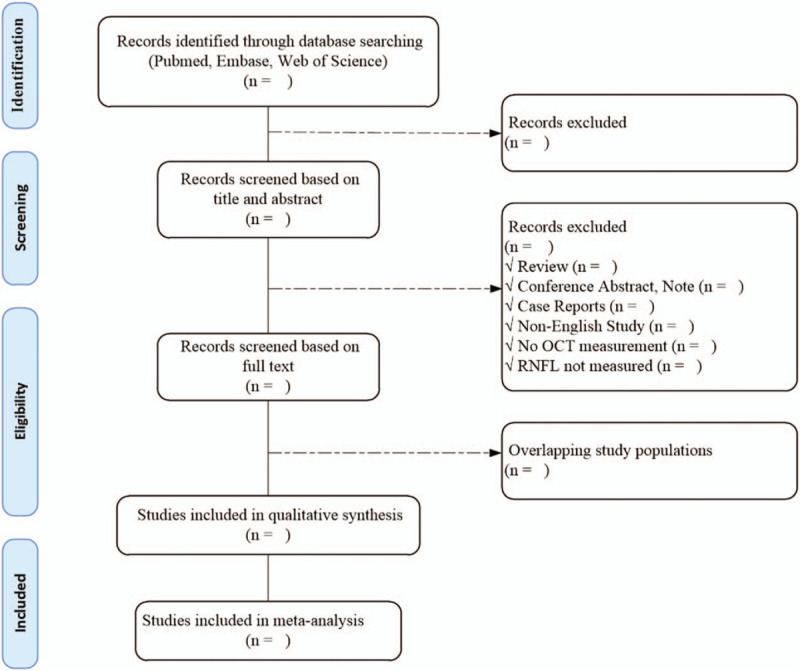
The PRISMA flow diagram of study selection process.

Data screening and extraction will be performed using Endnote X9 and Excel 2016. Two authors will check the title and abstract of the initially retrieved article to exclude duplicate and irrelevant research. Then the full text of the remaining articles will be read to further screen out the documents that meet the predetermined eligibility criteria. If there is a dispute between the 2 authors, a third researcher will join the discussion until consensus is reached. The process of study selection is fully provided in the following PRISMA flow diagram in Figure 1.

#### 2.3.2 Data extraction

Two reviewers will independently extract and fill out outcome measures for eligible studies in the excel data extraction form. The information extracted from the studies selected will include: study setting (first author, publication year), participants’ characteristics (age, gender, frequency of attacks, migraine disability assessment scores (MIDAS), duration of disease, and OCT model), and main outcomes.

### 2.4 Risk of bias assessment

The risk of bias for each eligible study will be evaluated by 2 reviewers using the Newcastle-Ottawa Scale (NOS).^[34]^ The tool contains eight assessment indicators, which are divided into 3 aspects: selection (4 items), comparability (1 item), and outcome (3 items). The scoring results will be presented on a table and we will assess the risk of bias in eligible studies.

### 2.5 Assessment of heterogeneity

The heterogeneity of data in each literature will be assessed by I^2^ test. If I^2^ ≤ 50%, the fixed effect model will be applied to synthesize the data, while I^2^ > 50% will be considered as large heterogeneity of the trial. The random effect model will be used, and the source of heterogeneity will be further analyzed by analysis, sensitivity analysis and regression analysis.

### 2.6 Statistical analysis

#### 2.6.1 Subgroup analysis

When the heterogeneity is high and there is sufficient data, we will conduct subgroup analysis based on patient characteristics, instrument type, and test results to obtain an objective conclusion, such as migraine with and without aura, different segmental RNFL, SD-OCT and TD-OCT.

#### 2.6.2 Sensitivity analysis

If necessary, we will perform sensitivity analysis to determine the robustness of the results, and to detect whether there are trials with high risk of bias accounting for a large proportion of the heterogeneity. If high-risk studies are deleted, a meta-analysis will be performed again, and the results will be discussed accordingly.

#### 2.6.3 Meta-regression analysis

Meta-regression analysis will be used to assess the impact of a series of influencing factors such as age, gender, disease duration, attack frequency, pain intensity, and intraocular pressure on the outcomes.

### 2.7 Publication bias

When more than 10 eligible trials are available for analysis, we will perform an Egger test using Stata 15 software to analyze potential publication bias.

### 2.8 Ethics and dissemination

No ethical review is required, as the data used in this study is extracted from published studies, which does not involve the personal data of the participants.

## 3 Discussion

Migraine, as the most common episodic neurological disorder, has become a global public health problem.^[35]^ OCT is a common non-invasive imaging technique for measuring the retina and RNFL.^[36]^ Altered RNFL thickness investigated by OCT in migraine patients has been reported by many studies. In 2015, a meta-analysis has synthesized the relevant literatures and concluded that the RNFL thickness of migraine patients with optical coherence tomography is lower than that of healthy controls.^[31]^ Since then, there have been many publications eligible for inclusion, which may change the conclusion of the previous meta-analysis. Therefore, we will re-screen and update the relevant articles, hoping to provide some directions for the early diagnosis of migraine by monitoring the RNFL changes in the future.

Overall, we will quantitatively analyze RNFL thickness differences between migraine patients and healthy controls. Assess the quadrant most affected by migraine and analyze the cause. We speculate that thinning of RNFL thickness may be more affected by migraine with aura. Occipital hemispheric vasospasm and subsequent reduction in blood flow in migraine with aura and hypoperfusion around the optic nerve head may lead to retinal ganglion cell death.^[37]^ In addition, RNFL measured by SD-OCT may be more relevant to migraine than TD-OCT., for the SD-OCT can detect more subtle damage to RNFL.^[38]^

## Author contributions

**Conceptualization:** XueLing Zhang, HongJie Zhang

**Data curation:** XueLing Zhang, ZhongQuan Yi

**Formal analysis:** ZhongQuan Yi, QinQin Liu

**Investigation:** QinQin Liu, XiaoGuang Lin

**Methodology:** QinQin Liu, ZhongQuan Yi

**Project administration:** PingLei Pan

**Software:** HongJie Zhang, ChaoChun Chen, RuYuan Cai

**Supervision:** QinQin Liu

**Writing – original draft:** XiaoGuang Lin, XueLing Zhang

**Writing – review & editing:** PanWen Zhao, PingLei Pan
